# Oganesson: A Noble Gas Element That Is Neither Noble Nor a Gas

**DOI:** 10.1002/anie.202011976

**Published:** 2020-10-22

**Authors:** Odile R. Smits, Jan‐Michael Mewes, Paul Jerabek, Peter Schwerdtfeger

**Affiliations:** ^1^ The New Zealand Institute for Advanced Study and the Institute for Natural and Mathematical Science Massey University (Albany) 0632 Auckland New Zealand; ^2^ Mulliken Center for Theoretical Chemistry University of Bonn Beringstr. 4 53115 Bonn Germany; ^3^ Nanotechnology Department Helmholtz-Zentrum Geesthacht Max-Planck-Straße 1 21502 Geesthacht Germany

**Keywords:** density functional theory, free energy calculations, melting and boiling processes, Monte Carlo simulations, oganesson

## Abstract

Oganesson (Og) is the last entry into the Periodic Table completing the seventh period of elements and group 18 of the noble gases. Only five atoms of Og have been successfully produced in nuclear collision experiments, with an estimate half‐life for 294118 Og of 0.69+0.64-0.22 ms.^[1]^ With such a short lifetime, chemical and physical properties inevitably have to come from accurate relativistic quantum theory. Here, we employ two complementary computational approaches, namely parallel tempering Monte‐Carlo (PTMC) simulations and first‐principles thermodynamic integration (TI), both calibrated against a highly accurate coupled‐cluster reference to pin‐down the melting and boiling points of this super‐heavy element. In excellent agreement, these approaches show Og to be a solid at ambient conditions with a melting point of ≈325 K. In contrast, calculations in the nonrelativistic limit reveal a melting point for Og of 220 K, suggesting a gaseous state as expected for a typical noble gas element. Accordingly, relativistic effects shift the solid‐to‐liquid phase transition by about 100 K.

## Graphical abstract

Is the latest addition to the periodic table an actual or a formal noble gas? Two complementary state‐of‐the‐art approaches reveal in unison that Og is a solid at ambient conditions. And with predicted melting and boiling points of 325±15 K and 450±10 K, not even particularly volatile.

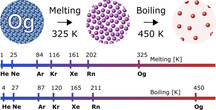

Six new elements (Nh, Fl, Mc, Lv, Ts and Og) have been added into the Periodic Table of Elements over the past 20 years, completing the 7p shell and the 7th period.^[2]^ These exotic short‐lived superheavy elements can only be created at a one‐atom‐at‐a‐time scale with production rates of one atom per week or even less. Experiments to explore their chemistry is thus very limited,[^3^, ^4^, ^5^, ^6^, ^7^] and only accurate computational approaches based on either wavefunction or density functional theory can give a detailed glimpse into their physical and chemical properties. These superheavy elements show very unusual behavior compared to their lighter congeners due to strong relativistic effects.[^8^, ^9^, ^10^] For example Cn and Fl are predicted to be chemically inert[^7^, ^11^, ^12^] due to the relativistic 7s shell contraction for Cn and the large spin‐orbit splitting of the 7p shell, resulting in a closed 7p_1/2_ shell for Fl.

In contrast to all other noble‐gas solids, Og was recently predicted to be a semiconductor.^[13]^ Further, the electron localization function for the Og atom shows a uniform electron‐gas‐like behavior in the valence region, accompanied by a large dipole polarizability.^[8]^ These findings indicate that for the interaction between Og atoms, 3‐body effects might become more important than for the lighter noble gases. Indeed, this was recently confirmed by calculations, which also revealed a stark increase in the many‐body interaction due to relativistic effects.^[14]^ Based on such a many body expansions derived rigorously from relativistic coupled cluster theory, the melting temperature of the noble gases from Ne to Rn were obtained through parallel tempering Monte Carlo (PTMC), resulting in deviations of not more than a few Kelvin compared to experimental results.[^15^, ^16^, ^17^] For a general review on rare gas solids we refer to ref. [18].

Considering the unusually strong attractive interaction in the Og dimer,^[14]^ one might speculate that Og is a solid at room temperature, although different extrapolations lead to contradictory results.[^19^, ^20^, ^21^] In order to resolve this long‐standing controversy, we employ two complementary approaches to calculate the melting temperature of Og. Firstly, we use PTMC simulations with direct sampling of the bulk using periodic boundary conditions, as well as magic number icosahedral clusters where the melting temperature is obtained from extrapolation to the bulk limit.[^15^, ^16^, ^17^] These PTMC calculations employ 2‐and 3‐body potentials derived from relativistic coupled‐cluster (CC) calculations.^[14]^ Secondly, to verify these results and moreover to determine the boiling point, we use thermodynamic integration (TI) based on relativistic dispersion‐corrected density‐functional theory to calculate absolute Gibbs energies of solid and liquid Og, while gaseous Og is modeled as non‐interacting (ideal) gas.[^7^, ^12^] Subsequently, linear extrapolation to the intersections between the solid, liquid and gaseous Gibbs energies eventually provides the melting and boiling points. A detailed description of the methods used can be found in the supplementary material.

We start with the discussion on the PTMC results for finite clusters in the canonical ensemble. Melting simulations were performed for Mackay icosahedral magic clusters of size N=13, 55, 147, 309, 561, 923 and 1415 atoms considering 2‐body interactions only, with additional simulations including 3‐body interactions up to a cluster size of *N*=923. Heat capacities of the icosahedral clusters as a function of the simulated temperature are shown in Figure 1. Here, the highest peak for a specific cluster size corresponds to the solid‐to‐liquid phase transition of the entire cluster, whereas smaller peaks are associated with structural transitions (so‐called pre‐melting).^[22]^ The bulk melting temperatures were determined by extrapolation of the finite cluster results to the bulk value with inverse cluster radius, equivalent to *N* ^−1/3^. 2‐body melting temperatures were obtained by extrapolation of the clusters *N*=147–1415 and 3‐body corrections were taken as the difference in melting temperature when extrapolating clusters of size *N*=147–923 including 2‐ versus 2+3‐body interactions, see Figure 1 for the extrapolation and Table 1 for a summary of the results.

**Figure 1:**
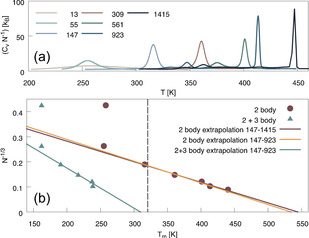
(a) Heat capacities for the finite icosahedral clusters, simulated with the RX2C 2‐body potential. (b) Extrapolation from the finite cluster to the bulk melting temperature. In maroon, the extrapolation to the bulk 2‐body melting temperature. The 3‐body correction is estimated as the difference between the extrapolated melting temperature of the 2‐body (in yellow) and the 3‐body corrected melting temperatures for the *N*=147–923 clusters (in green). The final 3‐body corrected melting temperature is indicated with the vertical grey line.

**Table 1:** Melting temperatures (in Kelvin) for the cluster and bulk simulations at the non‐relativistic (NR), scalar relativistic (SR) and fully relativistic (RX2C) level of theory using the 2‐ and 3‐body potentials from ref. [14]

		**NR**	**SR**	**RX2C**
finite cluster	*T*_m_ (2‐body)	244.7	332.8	544.4
	Δ*T*_m_ (3‐body)	−15.5	−63.0	−224.8
	*T*_m_ (2+3‐body)	229.1	269.8	319.7
				
periodic bulk	*T*_m_ (2‐body)	238.8	330.4	554.4
	Δ*T*_m_ (3‐body)	−18.2	−62.0	−230.5
	*T*_m_ (2+3‐body)	220.7	268.3	324.0

Let us now move to the results obtained for bulk cells with periodic boundary conditions, which are simulated in the isobaric‐isothermal ensemble at 1 atm pressure. Since the solid‐to‐liquid phase transition temperature is known to converge to the superheated melting temperature *T*_SH_ with increasing cell size, the melting temperatures extracted from the bulk simulations are corrected using the expression *T*_m_=*T*_SH_/1.231.[^17^, ^23^] Due to the high computational cost of the 3‐body corrections, this is accomplished in two steps: Firstly, for the largest cell (*N*=864) using the 2‐body potential, and secondly including the influence of 3‐body effects for a smaller (*N*=256) cell. The results of the periodic bulk and finite cluster calculations are collected in Table 1.

Inspection of Table 1 reveals excellent agreement between the periodic and cluster simulations at all levels. For the relativistic MP at the 3‐body level, they provide 324 K and 320 K, respectively. Relativistic effects significantly increase the cohesion between the atoms, which is due to strong increase of the attractive 2‐body interactions overcompensating a weaker increase of the repulsive 3‐body interactions compared to the non‐relativistic potential.

Accordingly, we find the relativistic contributions to the interaction potential to have a large influence on the melting transition. While calculations in the non‐relativistic limit show that Og would be a liquid or a gas at room temperature with a melting temperature of ≈220 K, as expected for a typical noble gas element, relativistic effects shift the solid‐to‐liquid phase transition by about 100 K, which is in equal parts due to scalar‐relativistic effects (48 K) and spin‐orbit coupling (56 K).

Concerning the many‐body decomposition, we find 3‐body contributions to be much larger compared to all other noble gases (Δ*T*_m_^3−body^ for Xe ≈20 K, for Rn ≈50 K).[^16^, ^17^] However, we do not expect 4‐body contributions to exert a significant influence since their contributions merely increase the cohesive energy by 1.4 percent. This is in contrast to the 3‐body contributions, which lowers the cohesive energy by 30 percent.^[14]^ Nonetheless, the 4‐body contributions are of attractive type and therefore the calculated melting temperature should be interpreted as a lower bound to the true melting temperature.

Having discussed the results of the PTMC calculations, let us now move to the free‐energy calculations employing TI. For this, we use spin‐orbit relativistic DFT with projector‐augmented wave (PAW) pseudo‐potentials as well as parameters for the DFT‐D3 dispersion correction introduced in previous work.[^12^, ^14^] To establish the relation between the different functionals and resulting potentials, Figure 2 compares the potential energy curves of solid Og obtained with the 4‐body potential derived from relativistic coupled‐cluster theory (CC) to that obtained with spin‐orbit relativistic DFT with the PBE‐D3, PBEsol and SCAN functionals.

**Figure 2:**
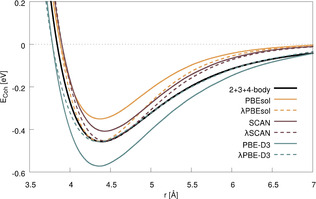
Comparison of the potential energy curves obtained with the 2+3+4 body CC potential and various density functionals along the stretching coordinate of fcc bulk Og. DFT curves scaled (λ) to the same potential depth as the 2+3+4‐body potential at equilibrium distance are also shown (dashed lines).

Since the melting point is very sensitive to the shape and depth of the potential energy surface for the bulk material, various density functionals can give quite different results.^[24]^ Hence, although at first glance SCAN provides the closest match to the CC cohesive energy, the functional with the best agreement concerning for the general shape of the potential curve is the dispersion‐corrected PBE‐D3. This becomes evident when the relative depth of the potential is corrected by linear scaling of the Hamiltonian (that is, interaction strength, all energies and forces) with a factor of *λ*=0.776. This can be seen as a showcase for the importance of using a dispersion correction with DFT, and we have thus selected PBE‐D3 for our study (a detailed description and discussion of this scaling is provided in ref. [25]).

After determining the equilibrium volume of the condensed phases at a simulation temperature of *T*=500 K, which corresponds to an effective temperature of *λ*T=388 K, we calculate their Gibbs energies via TI.^[25]^ For the liquid, represented by a 61 atom configuration, we begin from the non‐interacting atoms at the liquid equilibrium volume and integrate to the fully interacting liquid at the scalar‐relativistic DFT/PBE‐D3 level of theory. Subsequently, thermodynamic perturbation theory (TPT) is employed to include explicit spin‐orbit coupling and converge the numerical accuracy (details can be found in the supplementary). For the *fcc* solid represented by a 36 atom configuration, we start from the crystal at 0 K, calculate the Gibbs energy in the harmonic approximation, and eventually integrate to the scalar‐relativistic anharmonic solid. Similar to the liquid, spin‐orbit coupling and numerical convergence are included using TPT. Linear extrapolation of the liquid and solid Gibbs energies to their intersection provides a melting point of *T*_m_=425±14 K, which appears far too high compared to the PTMC results at first glance. However, this is due to the afore‐mentioned over‐binding of the PBE‐D3 functional, which can be corrected by means of *λ*‐scaling. This provides a value of *λT*_m_=330±11 K, and thus is in excellent agreement with the results of the PTMC method.

Since TI provides absolute Gibbs energies of the liquid, we can in addition to the melting temperature also determine the normal boiling point (NBP). To locate the intersection with the gas phase, gaseous Og is modeled as an ideal gas at normal pressure. This is a good approximation even at low temperatures, as evident from the negligible 2‐body virial correction ranging from 0.2 to 1 meV/atom from 500 to 200 K, which affects the NBP by less than 1 K. This approach predicts a NBP of Og is located at 453 K (562 K without *λ*‐scaling), meaning Og has an atypical large liquid range of 125 K for a noble gas.

Note that it was unfortunately not possible to obtain a detailed breakdown of the impact of relativistic effects in the DFT calculations due to technical issues. Specifically, while any consistent scalar‐relativistic treatment would require a time‐consuming scalar‐relativistic re‐parametrization of the DFT‐D3 correction, calculations in the non‐relativistic limit were prevented by convergence issues during the generation of the respective PAW pseudo‐potential.

Let us now discuss the calculated transition temperatures in the background of the previous literature. Grosse estimated the melting point by extrapolation of the critical temperature with the period number. On the basis that *T*_m_/*T*_c_=0.55 approximately holds for the noble gases up to Xe, the melting temperature of Og was estimated as 258 K.[^19^, ^26^] An extrapolation of the critical temperature with atom number *Z* would have perhaps been a better choice and leads to a melting temperature of 360 K. However, there is no theoretical justification for such linear relations. To see whether the melting temperature for Og follows the noble gas melting trend or not, one must first understand which quantity correlates with the melting temperature based on theoretical foundations rather than empirical observations.

Recently, we have shown that for a system with interaction strength scaled by a factor of *λ*, also the melting temperature increases by a factor *λ*, which has already been applied to correct the results of the TI above. Thus, the melting point directly correlates with the interaction energy, i.e. [Eq. 1]

(1) $$k_{B}T_{m}=f_{m}D_{e}=f_{m}f_{LJ}E_{coh}=\gamma E_{coh}$$

For example for a Lennard‐Jones system *γ*=12.16, as obtained from our PTMC simulations of an ideal LJ system in good agreement with previous results,[^27^, ^28^, ^29^, ^30^, ^31^, ^32^] and *f*_LJ_=0.116 obtained from Lennard‐Jones‐Ingham coefficients for the *fcc* crystal.^[33]^

Upon scaling of the 2‐body potentials (or equivalently the 2‐body cohesive energy curve) of the noble gases, these are all of near identical shape.^[14]^ The 2‐body melting point of Og is therefore expected to follow the linear scaling trend between melting point and cohesive energy, as set by the lighter congeners. Indeed, the noble gas melting temperatures obtained by simulation with the 2‐body potentials follow the predicted linear trend and, irrespective of the level of relativistic treatment, the 2‐body melting temperatures of Og fall on this line, as shown in Figure 3. For the lighter noble gas elements, the 3‐body and higher‐order contributions to the potential are small, such that the experimental melting temperatures as a function of cohesive energy also fall on the interpolated line obtained from the 2‐body scaling. However, the potential energy surface is altered significantly as 3‐body contributions in the heavier noble gases are enhanced by relativistic effects in the heavier noble gases, changing the pre‐factor γ in the equation above. As a consequence, the 3‐body corrected melting temperatures (and consequently the experimental melting temperatures) deviate substantially from the interpolated line for Og at a relativistic level of theory.

**Figure 3:**
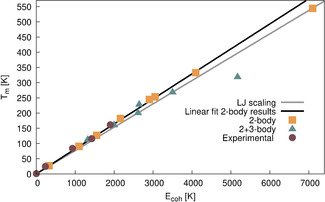
Melting points of the noble gases obtained with the PTMC periodic simulations plotted against their respective cohesive energy. In yellow squares the 2‐body melting temperatures plotted against their cohesive energies, with the black line showing the linear fit. The grey line shows the LJ potential scaled line. In green triangles the 3‐body corrected values and in maroon circles the experimental values.

Regarding the boiling point, Nash gave an estimation from extrapolation between the NBPs of the noble gases and their atomic polarizabilities to lie between 320–380 K (according to the same principle the NBP of Rn was estimated at 178–221 K,^[21]^ in agreement with the experimental value of 211 K^[16]^). Rescaling this estimate based on the latest high‐level value for the polarizability of *α*=58±6 a.u.^[14]^ leads to a NBP between 360–420 K. This is in contrast to our TI‐based result of 450±2 K, which moreover translates into an atypical large liquid range of almost 125 K (cf. Rn 9.5 K). Although this result might appear surprising and can not be confirmed by the PTMC approach, it should be pointed out that the employed Gibbs energy based approach has recently been tested for a representative set of elements of the periodic table, for which it provided boiling points in excellent agreement with experimental references with <2 % mean absolute deviation, and <1 % deviation for Xe.^[25]^ Based on the deviation in this comprehensive test, we provide a final estimate of 450±10 K for the NBP. In fact, the large liquid range can be rationalized by the unusual large 3‐body effect for Og compared to other noble gases, and moreover by the fact that Og was recently predicted to be a semiconductor.^[13]^ Semiconductors are known to have a larger temperature range for the liquid phase compared to the noble gases. Nevertheless, future work should include more accurate ab‐initio potential energy surfaces for the 3‐ and 4‐body contributions to confirm our predictions from DFT, which will be computationally challenging.

Finally, having discussed the phase transitions, let us move to the density of Og. From the periodic 3‐body corrected PTMC simulation, we obtain before melting starts a solid density of $\rho_{s}^{319K}$ =7.2 g cm^−3^, which decreases to $\rho_{l}^{327K}$ =6.6 g cm^−3^ for the liquid phase. The spin‐orbit relativistic PBE‐D3 calculations provide densities of $\rho_{s}^{390K}$ =7.38 g cm^−3^ and $\rho_{l}^{390K}$ =7.10 g cm^−3^ for solid and liquid Og, respectively. Applying the many‐body potentials including lattice vibrations to the *fcc* lattice, we obtain a nearest neighbor distance of *r*_nn_=4.396 Å, giving a density for ^294^Og of $\rho_{s}^{0K}$ =8.126 g cm^−3^ using the recommended isotopic mass of M_a_=294.214 amu. Grosse et al. estimated the density of Og by linear extrapolation of the atomic volume of the lighter noble gases as a function of period number using an atomic mass of 314 u. This resulted in a bulk density of $\rho_{s}^{0K}$ =6.29 g cm^−3^ and a density of the liquid at the melting point of $\rho_{l}^{256K}$ =4.92 g cm^−3^.[^19^, ^26^] Rescaling to an atomic mass of 294.214 u and extrapolating with the Z value instead, provides densities of $\rho_{s}^{0K}$ =5.89 g cm^−3^ and $\rho_{l}^{256K}$ =4.61 g cm^−3^ for the solid and liquid phase, respectively, and thus much lower than our predictions, as expected for such empirical estimates.

In summary, we have calculated the melting temperature of Og by means of parallel‐tempering Monte Carlo (PTMC) simulations based on an ab‐initio potential derived from high‐level relativistic coupled‐cluster theory, and through thermodynamic integration (TI) based on relativistic density‐functional theory. In excellent agreement with each other, these complementary approaches predict melting points of 324 K (Periodic PTMC), 320 K (Cluster PTMC) and 330 K (TI), which we combine to a final estimate of 325±15 K. Accordingly, we conclude that Og is a solid at ambient conditions. Moreover, based on the absolute Gibbs energy obtained via TI, we predict a NBP of 450±10 K, meaning that Og exhibits a large liquid range of 125 K. Although the large liquid range as well as the solid aggregate state are rather unusual for a noble gas element, they fall into place beside a series of further atypical properties. Altogether, this raises the question if Og should still be seen as a noble gas element, or if that title should be handed to the Group 12 element copernicium.^[12]^ Concerning periodic trends, we observe that the results obtained for the melting point of Og with the PTMC method using a hypothetical 2‐body potential limited to 2‐body interactions are in line with the lighter congeners. Only through inclusion of 3‐body effects, which are mostly of relativistic nature and thus larger for Og than for the lighter noble gases, a breakdown of periodic trends and relations can be observed.

## Conflict of interest

The authors declare no conflict of interest.

## Supporting information

As a service to our authors and readers, this journal provides supporting information supplied by the authors. Such materials are peer reviewed and may be re‐organized for online delivery, but are not copy‐edited or typeset. Technical support issues arising from supporting information (other than missing files) should be addressed to the authors.

Supplementary
