# Supplementary material for: Oganesson: A Noble Gas Element That Is Neither Noble Nor a Gas
Source: Angew Chem Int Ed Engl. 2020 Oct 22;59(52):23636–40. doi: 10.1002/anie.202011976 (PMC7814676; doi:10.1002/anie.202011976)
Supplement: Supplementary file 1 — Supplementary [file ANIE-59-23636-s001.pdf]

Angewandte Chemie
Eine Zeitschrift der Gesellschaft Deutscher Chemiker

Supporting Information

Oganesson: A Noble Gas Element That Is Neither Noble Nor a Gas

Odile R. Smits, Jan-Michael Mewes,* Paul Jerabek,* and Peter Schwerdtfeger**

anie_202011976_sm_miscellaneous_information.pdf

This supplementary material contains details of the two approaches employed to obtain the melting temperature of oganesson. We start with a description of the Monte Carlo melting simulation method and provide all the relevant melting results. Then, details on the thermodynamic integration method are given.

MONTE CARLO MELTING SIMULATIONS

Similar to our previous work on rare gases, we adapt a parallel tempering Monte Carlo scheme to obtain the inner energy as a function of temperature.[1–3] Here, a brief summary of the method is given. All remaining details of the Monte Carlo melting formalism can be found in Refs.[2,3].

The Monte Carlo melting simulations were performed at three different levels of relativistic treatment, non-relativistic (NR), scalar relativistic (SR) and fully relativistic (RX2C). Two different techniques to obtain the melting temperature are used; by direct determination of the melting temperature of the bulk and from finite clusters where the melting temperature is obtained from extrapolation of smaller cluster sizes to the bulk value.

The temperature at which the solid-liquid phase transition occurs can be identified from the sudden increase in the inner energy, which corresponds to the temperature where the heat capacity curve has a peak. The heat capacities, $C_v(T)$ and $C_p(T)$, for the cluster and periodic simulations respectively, are obtained from the relations

$$C_v(T) = \left.\frac{\partial U(T)}{\partial T}\right|_V = \frac{\langle U(T)^2\rangle - \langle U(T)\rangle^2}{k_B T^2} \quad (1)$$

$$C_p(T) = \left.\frac{\partial H(T)}{\partial T}\right|_P = \frac{\langle H(T)^2\rangle - \langle H(T)\rangle^2}{k_B T^2} \quad (2)$$

where the enthalpy is given by $H(T) = U(T) + PV$.

For each simulated temperature three million MC cycles were performed, and statistical data was collected from the last one million cycles. During each MC cycle, *N* configurations are generated by the displacement of a randomly chosen atom, where *N* is the number of atoms in the simulation cell/cluster. The acceptance criterion for moving the atoms is based on the Boltzmann criterion.

In total, 32 temperature trajectories were propagated simultaneously spanning the temperature interval of the melting transition. Exchanges of configurations are attempted of near-lying temperatures according to the parallel tempering method in order to overcome ergodicity problems and improve convergence. That is, an exchange is attempted between two neighbouring temperature configurations with a 10 % probability and an acceptance criteria based on the Boltzmann distribution. The statistical quantities such as the inner energy $U(T)$ and the heat capacity at constant volume, $C_v(T)$ (for finite cluster simulations), and volume $V(T)$, the enthalpy $H(T)$, and the heat capacity at constant pressure, $C_p(T)$ (for periodic simulations), were calculated using the two-body ELJ and three-body EATM potentials.

All relevant quantities are obtained as a continuous function of temperature *T* from the simulation data using the histogram re-weighting technique. In the following, specific details for the cluster and periodic simulations are given.

THE INTERACTION POTENTIAL

The total interaction potential, $E_{int}(N)$, for an N atomic system can be expanded into sums over all possible two-body, $E^{(2)}(r_{ij})$, three-body, $E^{(3)}(r_{ij}, r_{ik}, r_{jk})$, and higher-order, $E^{(N)}(N)$, contributions,

$$\begin{aligned} E_{int}(N) &= \sum_{n=2}^{N} E^{(N)}(N) \\ &= \sum_{i<j}^{N} E^{(2)}(r_{ij}) + \sum_{i<j<k}^{N} E^{(3)}(r_{ij}, r_{ik}, r_{jk}) + \cdots \end{aligned}$$

where r_{ij} is the distance between atoms *i* and *j*. The many-body expansion converges rapidly at normal pressures and temperatures for the rare gas elements including oganesson. [4]

In earlier work, data points for two-body and equilateral three-body potentials were constructed using nonrelativistic, scalar-relativistic, and relativistic coupled-cluster theory.[4] The data points for the two-body potential curves were pointwise fitted to an extended Lennard-Jones (ELJ) potential by a linear least-squares procedure

$$E_{ELJ}^{(2)}(r_{ij}) = \sum_{k=0}^{N} C_{k+6}\, r_{ij}^{-(k+6)}$$

where r_{ij} is the internuclear distance between atoms *i* and *j* and C_{k+6} are the fitting parameters.[5]

The three-body corrections, $E^{(3)}(r_{ij}, r_{ik}, r_{jk})$, were fitted to a computer-efficient extended Axilrod-Teller-Muto (EATM) potential[6,7]

$$E_{EATM}^{(3)}(r_{ijk}) = f_\theta[C_{EAT}R_g^{-9} + (A_0 + A_2R_g^2 + A_4R_g^4 + A_6R_g^6)e^{-\alpha R_s}$$

with

$$f_\theta = (1 + 3\,\cos\theta_i\,\cos\theta_j\,\cos\theta_k), \qquad R_g = (r_{ij}r_{ik}r_{jk})^{1/3}$$

and

$$R_s = r_{ij} + r_{ik} + r_{jk}$$

where the angle-dependence comes from the dominant triple-dipole term, but the radial dependence contains all higher moments. The computed spin-orbit contributions to the three-body potential are fitted to the exponential form

$$E_{SO}^{(3)}(r_{ijk}) = f_\theta[a + bR_g + (cR_g)^2\,]e^{-dR_s}$$

such that the total three-body two-component relativistic (RX2C) potential is described by

$$E_{EATM,SO}^{(3)}(r_{ijk}) = E_{EATM}^{(3)}(r_{ijk}) + E_{SO}^{(3)}(r_{ijk})$$

The corresponding parameters can be found in Table 2 in Ref [4].

CLUSTER SIMULATIONS

For our simulations, Mackay icosahedral clusters are chosen as initial structures for the solid state which are characterized by *k* complete shells of atoms around a central atom. To keep the clusters from evaporating, a hard sphere is defined with a radius of one diatomic equilibrium distance larger than the radius of the icosahedral cluster. For this type of simulation, the hard-sphere volume, *V*, is thus held constant during the MC simulation, while the pressure is allowed to vary. Melting simulations were performed for Mackay icosahedral clusters of size *N* = 13, 55, 147, 309, 561, 923 and 1415 atoms considering two-body interactions only, and additional simulations were performed considering two+three-body interactions up to clusters containing 923 atoms.

Two-body interactions were considered over the entire sphere, whereas for the three-body interaction a cut-off radius of r_c =2σ was employed, where σ is the position of the maximum of the three-body EATM potential.

Heat capacity curves are shown in Figs. 1-3 and melting temperatures are collected in Table 1. By plotting the melting temperatures as a function of $N^{-1/3}$, as shown in Fig. 4, the melting temperatures exhibit a linear trend at larger *N* values and can be extrapolated to the bulk value. The two smallest clusters of size *N* = 13 and *N* = 55 deviate from this line as to be expected [see the discussion below Eq. (16) in Ref.[3] and are therefore not considered in the extrapolation. Two-body melting temperatures were therefore obtained by extrapolation of the clusters *N* = 147 to 1415 and three-body

corrections were taken as the difference in melting temperature when extrapolating clusters of size N = 147 to 923 including two- versus two+three-body interactions. The melting temperatures obtained by extrapolation are collected in Table 2.

PERIODIC SIMULATIONS

For the periodic simulations the melting temperature was determined by direct sampling of the bulk using cells with periodic boundary conditions in the isobaric-isothermal ensemble at 1 atm pressure. The melting simulations were performed for cells with N = 32, 108, 256, 500 and 832 atoms considering two-body interactions only, and additional simulations were performed with two+three-body interactions and cells containing up to 256 atoms.

The total two-body interaction energy , $E^{(2)}(r_{ij})$, was determined by summing over all pairwise interactions up to a spherical cut-off distance of $r_c = \frac{1}{2}L$ where L is the length of the simulation cell. Three-body contributions were calculated up to a cut-off of $r = 2\sigma$, where σ is the position of the maximum of the three-body EATM potential.

It was demonstrated in our earlier work that the solid-to-liquid phase transition converges with cell size to the superheated melting temperature,[1–3] and we observe the same behaviour for our oganesson simulations. We therefore adapt the superheating correction factor [8]

$$\frac{T_{SH}}{T_m} = 1 + \frac{\ln 2}{3} \approx 1.231. \tag{3}$$

The heat capacities of the different cell sizes are shown in Fig. 5-7 and the for superheating corrected melting temperatures are collected in Table 3.

The two-body melting temperatures were taken from the N = 864 cell. Constraint by the high computational cost, the three-body corrections are estimated from the difference in melting temperature between the two-body and the three-body corrected melting temperatures of the N=256 cell.

MELTING RESULTS

Upon correction for superheating we achieve excellent agreement between the periodic and cluster simulations. Melting temperatures of the non-relativistic, scalar relativistic and spin-orbit relativistic simulations are provided in Table 4.

As mentioned in the main paper, Grosse obtained an estimate for the melting temperature of oganesson by extrapolation with period number of the lighter noble gases and their respective critical temperature.[9,10] Fig. 10 shows the melting temperatures of the noble gases as a function of atomic mass. As evident from this figure, an extrapolation with mass number would have led to a better estimate. However, keep in mind that there is no theoretical justification for this apparent linear behaviour. As argued in the main paper, there is a linear relation between the cohesive energy and melting temperature, but only if the potential energy surface is of identical shape. The cohesive energies and melting temperatures of the noble gases are given in Table 5. A deviation of the ratio between the melting temperature and cohesive energy of the three-body corrected oganesson at spin-orbit level of theory compared to the other noble gases is clearly noticeable.

DFT-BASED THERMODYNAMIC INTEGRATION/GIBBS ENERGY CALCULATION

Solid – The Gibbs energy of the solid is calculated for a 36 atom *fcc* super-cell at 500 K (effective temperature λT = 388 K) with a Langevin thermostat and a timestep of 8 fs. The calculation consists of four main steps, the first three of which are conducted in the scalar-relativistic approximation (LSORBIT = FALSE) and with reduced numerical precision (400 eV cut-off, Γ-point approximation, PRECISION=NORMAL) to enable DFT-MD simulations. In the first step, the electronic energy and ionic frequencies of the super-cells are calculated at the equilibrium volume in the scalar-relativistic limit. For the finite-differences calculations, the numerical precision is increased by setting Precision=Accurate and LREAL=False in the INCAR file to avoid aliasing errors. Moreover, since we noted a large influence of the size of the displacements (POTIM) on the frequencies, four displacements are considered for each atom (NFREE=4) instead of the default of two. By setting ISPECIAL in the INCAR file to -1, this calculation writes the full harmonic Hessian for the system to a file DYNMATFULL, which will be needed later. In the second step, the vibrational contributions to the Gibbs energy are calculated in the harmonic approximation based on the previously calculated phonon frequencies. These calculations are carried out with the Phonopy program using a very fine (16*16*16) k-point mesh.[11] Combining the electronic energy from the first step (-0.3757 eV/atom) with the vibrational contributions from the second step (-0.3720 eV/atom), we obtain the Gibbs energy of solid Og in the (scalar-relativistic) harmonic approximations (0.7477 eV/atom). However, at the solid near the melting point, anharmonic contributions can be significant and are thus calculated explicitly in a third step. To obtain these, a thermodynamic integration (TI) is conducted from the harmonic crystal (forces calculated in step 1) to scalar-relativistic DFT. The integral over the coupling constant, which interpolates between harmonic and DFT forces (using the SCALEE keyword and the DYNMATFULL file generated in step 1), is evaluated numerically with a three-point Gauss-Legendre rule (λ= [0.1127; 0.5000; 0.8873]), with about 10,000 steps (80 ps) at each point (10 ps equilibration + 70 ps production). This provides an anharmonic correction of 6.41 meV/atom (total of -0.7413 eV/atom). While this might appear small contribution at first glance, it has a large influence on the melting point, which moves down by 70 K upon inclusion of these anharmonic effects. In the fourth and final step, the influence of spin-orbit coupling in the valence space and increased numerical precision are accounted for. For this, 10-20 configurations from the DFT-MD simulation at the solid equilibrium volume are taken and recalculated with a higher energy cut-off (400 to 600 eV, PRECISION NORMAL to ACCURATE, contribution 1.0 meV/atom), a finer k-point grid (at 600 eV, Γ to 2^3, contribution -0.0115 eV/atom, 2^3 to 3^3, contribution < 0.1 meV/atom), and including spin-orbit coupling (at 600 eV and 2^3 k-points, contribution -0.1702 meV/atom). Altogether, this provides a final Gibbs energy of the solid of -0.9220±0.0004 eV/atom, a final internal energy of 0.4427±0.0004 eV/atom, and this a total entropy of 0.9587±0.0008 meV/atom/K.

Liquid – The Gibbs energy of the liquid is calculated for a 61 atom liquid configuration at 500 K (effective temperature λT = 388 K) with a Nose-Hover thermostat and a timestep of 8 fs. Thermodynamic integration is conducted from a reference of non-interacting point masses (ideal gas) to the DFT liquid. For the non-interacting reference, we calculate a Gibbs energy of -0.6226 eV/atom (at the liquid equilibrium volume). Since the contribution from TI is much larger than for the solid, the integral is evaluated with a 9-point Gauss-Lobatto rule. Moreover, to avoid the singularity at λ=0 and increase the density of quadrature points near the non-interacting limit, the integral is transformed as described in ref.[12]. This introduces a parameter ϰ, which guides the mapping of the quadrature points, and for which we use a value of 0.6 after careful testing. The resulting quadrature points are λ = [1.000; 0.8794; 0.6440; 0.3835; 0.1768; 0.0572; 0.0105; 0.0006]. At each of the points, about 20,000 steps (160 ps) of NVT DFT-MD are accumulated, which provides sufficiently converged average internal energies. The contribution from TI amounts to -0.1364 eV/atom, giving a total Gibbs energy of -0.7590 eV/atom. Similar to the solid, TI is followed by thermodynamic perturbation theory to include spin-orbit coupling and converge the numerical parameters. For this, 10-20 configurations from the DFT-MD at the final liquid volume are taken and recalculated with a higher energy cut-off (400 to 600 eV, PRECISION NORMAL to ACCURATE, contribution 0.5 meV/atom), a finer k-point grid at 600 eV (Γ to 2^3, contribution -0.0029 eV/atom, 2^3 to 3^3, contribution < 0.1 meV/atom), and including spin-orbit coupling (at 600 eV and 2^3 k-points, contribution -0.1660 meV/atom). Altogether, this provides a final Gibbs free of the liquid of -0.9274±0.0007 eV/atom, a final internal energy of 0.4121±0.009 eV/atom, and thus a total entropy of 1.0305±0.0024 meV/atom/K.

Gas – The free energy of the gas phase is calculated analytically using the ideal-gas law plus a 2-body virial correction based on a Lennard-Jones potential as described in ref.[13].

Errors are in the simulation averages are calculated using block-averaging (block-size in the solid simulations ~ 100-150, in the liquid simulations 150-200 steps) and traced using Gaussian error-

propagation. This provides a statistical error in the melting point of ±11 K, while the error in the boiling point is much smaller with ±2 K. The smaller error in the BP is a result of the steeper intersection between the respective Gibbs energy curves.
The more conservative errors of the final estimates given in the conclusion (±15 K for the BP and ±10 K for the MP) are derived (i) from the agreement between PTMC and TI for the melting point, and (ii) from the mean-average deviation of the approach in a recent comprehensive test.[14]

DENSITIES

Monte-Carlo Simulations: Fig. 8 shows the temperature dependence of the densities and in particular the densities shortly before and after melting are listed in Table 6. Two-body densities are extracted from the N = 864 simulation cells and three-body corrected values are extracted from the N = 256 simulation cell. As a reference, Table 7 contains results from solid-state calculations for the fcc crystals using the two-body ELJ and three body EATM terms as well as the classical four-body Drude term and the two-body vibrational contributions.[15] Fig. 9 shows the densities of the solid at 0 K obtained from lattice sums, the density of the solid just before melting and the density of the liquid just after melting for the noble gases. Also shown are the densities of oganesson and radon as estimated by extrapolation of atomic volume with period number, estimations obtained by Grosse.[9,10] Just as for the estimate of the melting temperature, an extrapolation with mass number would have led to a better estimate.

DFT Simulations: Equilibrium volumes of the solid and liquid were determined for 36-atom and 61-atom configurations, respectively. The simulations are conducted at 500 K (effective temperature 388 K) in the canonical (NVT) ensemble with an 8 fs timestep and a Langevin thermostat for the solid and a Nose-Hover thermostat for the liquid. The pressure is averaged over several thousand steps (until the variance is smaller than 0.2 kBar) at slightly different volumes at a scalar-relativistic level, with an energy cut-off of 400 eV and in the γ-point approximation. The influence of Pulay stress, a finer k-point grid and spin-orbit coupling are including by conducting single-point calculations for 10 equidistant snapshots from the trajectory with a cut-off of 600 eV, a 2^3 k-point grid at a spin-orbit relativistic level. This is possible since the shifts at the different snapshots are very similar (variance < 0.05 kBar). The corrected pressures at each volume are fitted with a second-order polynomial and interpolated to the x-intersection (p = 0).
For the solid, this provided a final equilibrium volume of 66.11 A^3/atom (density 7.38 g/cm^3, residual pressure -0.4 kBar). For the liquid, we obtained 68.77 A^3/atom (7.10 g/cm^3, residual pressure -0.5 kBar).

REFERENCES

[1] E. Pahl, F. Calvo, L. Koči, P. Schwerdtfeger, *Angew. Chemie Int. Ed.* **2008**, *47*, 8207–8210.
[2] O. R. Smits, P. Jerabek, E. Pahl, P. Schwerdtfeger, *Angew. Chemie - Int. Ed.* **2018**, *57*, 9961–9964.
[3] O. R. Smits, P. Jerabek, E. Pahl, P. Schwerdtfeger, *Phys. Rev. B* **2020**, *101*, 104103.
[4] P. Jerabek, O. R. Smits, J. Mewes, K. A. Peterson, P. Schwerdtfeger, *J. Phys. Chem. A* **2019**, *123*, 4201–4211.
[5] P. Schwerdtfeger, N. Gaston, R. P. Krawczyk, R. Tonner, G. E. Moyano, *Phys. Rev. B* **2006**, *73*, 64112.
[6] L. W. Bruch, I. J. Mcgee, I. J. Mcgee, **1973**, *59*, 409.
[7] P. Schwerdtfeger, A. Hermann, *Phys. Rev. B - Condens. Matter Mater. Phys.* **2009**, *80*, 1–5.
[8] A. B. Belonoshko, N. V Skorodumova, A. Rosengren, B. Johansson, *Phys. Rev. B* **2006**, *73*, 12201–12203.
[9] A. V Grosse, *J. Inorg. Nucl. Chem.* **1964**, *26*, 1801–1809.
[10] A. V. Grosse, *J. Inorg. Nucl. Chem* **1965**, *27*, 509–519.
[11] A. Togo, I. Tanaka, *Scr. Mater.* **2015**, *108*, 1–5.
[12] F. Dorner, Z. Sukurma, C. Dellago, G. Kresse, *Phys. Rev. Lett.* **2018**, *121*, 195701.
[13] J.-M. Mewes, O. R. Smits, G. Kresse, P. Schwerdtfeger, *Angew. Chemie Int. Ed.* **2019**, *58*, 17964–17968.
[14] J.-M. Mewes, O. R. Smits, *Phys. Chem. Chem. Phys.* **2020**, DOI chemrxiv.12301934.v3.
[15] P. Schwerdtfeger, R. Tonner, G. E. Moyano, E. Pahl, *Angew. Chem. Int. Ed.* **2016**, *55*, 12200–12205.

Table 3. Melting temperatures in Kelvin for the simulated rare gas clusters extracted from the heat capacity curves.

	N	13	55	147	309	561	923	1415
**Og NR**								
	2b	107.73	111.36	137.46	154.00	175.86	183.98	194.00
	2+3 b	101.99	106.00	132.36	147.68	166.56	174.63	
**Og SR**								
	2b	151.07	151.58	187.0	214.3	240.8	248.08	266.00
	2+3 b	128.5	130.83	157.14	175.91	196.49	204.7	
**Og RX2C**								
	2b	257.93	255.07	316	360.76	401.09	413.49	440.30
	2+3 b	162.16	162.17	190.82	216.96	237.5	238.5	

Table 3. Melting temperatures in Kelvin obtained by linear extrapolation of $N^{-1/3}$ to the bulk melting temperature.

	N	2b	2 + 3 body	Δ (2b - 3b)
**Og NR**				
	[147-1415]	244.65	-	-
	[147-923]	242.38	226.86	15.51
**Og SR**				
	[147-1415]	332.83	-	-
	[147-923]	326.79	263.75	63.04
**Og RX2C**				
	[147-1415]	544.40	-	-
	[147-923]	534.34	309.59	224.75

Table 3. Melting temperatures, corrected for superheating, in Kelvin for the simulated cells with periodic boundary conditions.

	N	32	108	256	500	864
**Og NR**						
	2 body	228.81	231.09	237.61	239.20	238.83
	2 +3 body	221.67	213.06	219.43	-	-
	Δ(2b-3b)	7.14	18.03	18.18	-	-
**Og SR**						
	2 body	320.07	315.29	328.19	327.94	330.35
	2 +3 body	275.94	259.15	266.18	-	-
	Δ(2b-3b)	44.13	56.14	62.01	-	-
**Og RX2C**						
	2 body	540.56	531.16	555.00	553.48	554.44
	2 +3 body	357.21	318.55	324.53	-	-
	Δ(2b-3b)	183.35	212.61	230.46	-	-

Table 3. Final melting temperatures in Kelvin for the periodic and cluster simulations.

		Periodic	Cluster
**Og NR**			
	2b	238.83	244.65
	Δ(2b-3b)	18.18	15.51
	T_m	220.65	229.14
**Og SR**			
	2b	330.35	332.83
	Δ(2b-3b)	62.01	63.04
	T_m	268.34	269.79
**Og RX2C**			
	2b	554.44	544.4
	Δ(2b-3b)	230.46	224.75
	T_m	323.98	319.65

Table 5. Cohesive energies, E_{min}, and melting temperatures, T_m, for the nobles gases, both in Kelvin. The 2+3 body cohesive energies are obtained from solid state calculations performed with SAMBA.

		E_{min} [K]	T_m [K]	E_{min}/ T_m
LJ		8.22	0.676	12.16
Ne	2b	326.20	26.9	12.13
Ar	2b	1095.72	90.6	12.09
Kr	2b	1521.05	126.85	11.99
Xe	2b	2161.25	182.24	11.86
Rn	2b	3052.07	253.32	12.05
Og NR	2b	2904.27	244.65	11.87
Og SR	2b	4095.90	332.83	12.31
Og SO	2b	7099.04	554.4	12.80
Kr	2+3b	1318.70	113.72	11.60
Xe	2+3b	1995.27	161.58	12.35
Rn	2+3b	2613.96	201.64	12.96
Og NR	2+3b	2643.89	229.14	11.54
Og SR	2+3b	3500.57	269.79	12.98
Og SO	2+3b	5170.03	319.65	16.17
Ne	Exp.	232.51	24.56	9.47
Ar	Exp.	928.74	83.85	11.08
Kr	Exp.	1422.53	115.79	12.29
Xe	Exp.	1898.34	161.35	11.76

Table 6. Densities, in g/cm^3, obtained from the PT MC simulations. In brackets the temperature at which the density is extracted.

	solid		liquid	
	$E^{(2)}$	$+E^{(3)}$	$E^{(2)}$	$+E^{(3)}$
Og NR.	5.4 (235 K)	5.4 (218 K)	4.5 (240K)	4.4 (223 K)
Og SR.	7.0 (326 K)	6.8 (258 K)	6.0 (333K)	5.9 (267 K)
Og RX2C	8.0 (550 K)	7.2 (319 K)	7.0 (560K)	6.6 (327 K)

Table 7. Density, in g/cm^3, for the fcc crystals of 294-Og obtained with the program package SAMBA. ZPE-H denotes zero-point harmonic vibrational correction within the harmonic Einstein approximation for the two-body term only, ZPV-AH is the corresponding anharmonic correction from first-order perturbation theory.

Density [g/cm^3]	Og. NR	Og. SR	Og. RX2C
$E^{(2)}$	6.256	8.156	9.348
+ ZPV-H	6.222	8.114	9.311
+ ZPV-AH	6.221	8.114	9.311
+ $E^{(3)}$	5.948	7.757	8.059
+ $E^{(4)}$	5.954	7.787	8.126

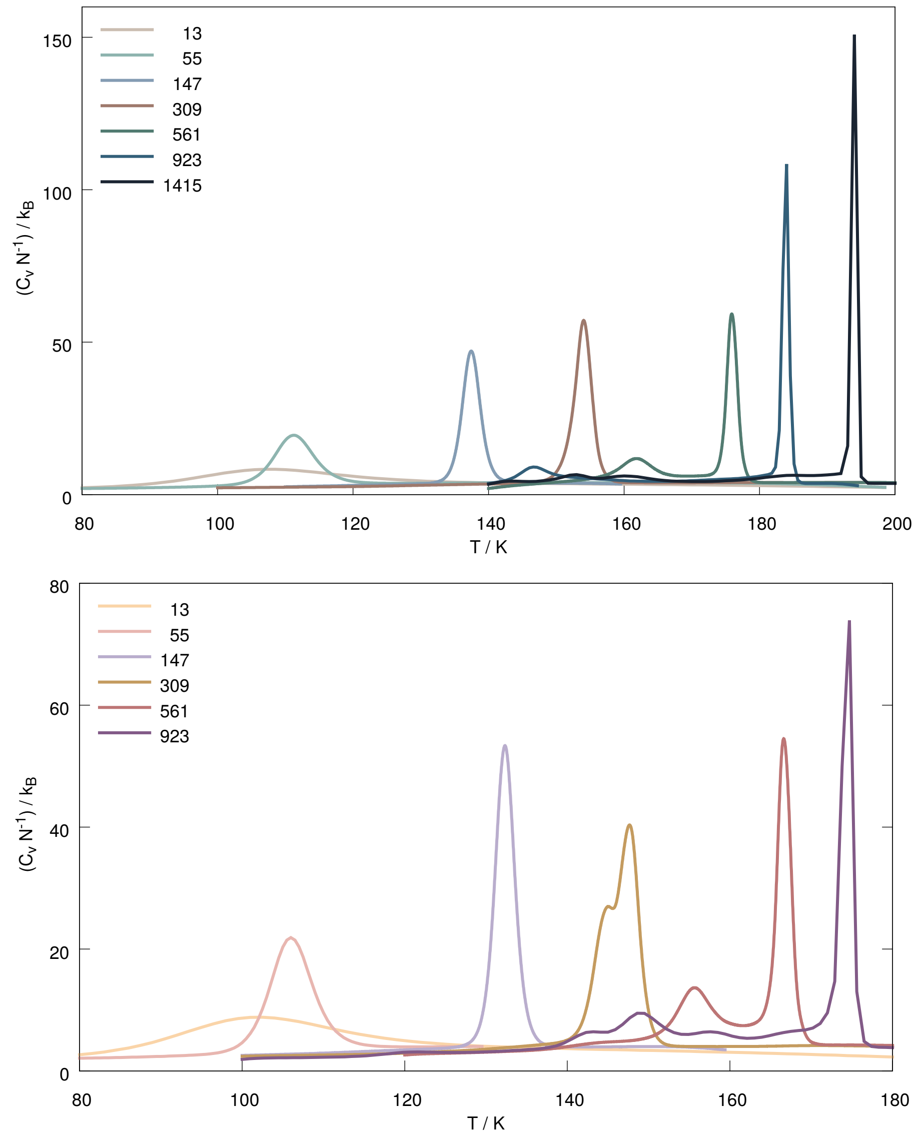

Figure 1. Heat capacities per atom as a function of temperature for simulations at constant volume with the Mackay icosahedral clusters up to 1415 atoms for oganesson at the non-relativistic (NR) level of theory. Top, heat capacity curves obtained considering 2-body interactions only, using the ELJ potential. Bottom, obtained heat capacities considering 2- and 3-body interactions, computed with the ELJ and EAT potentials respectively.

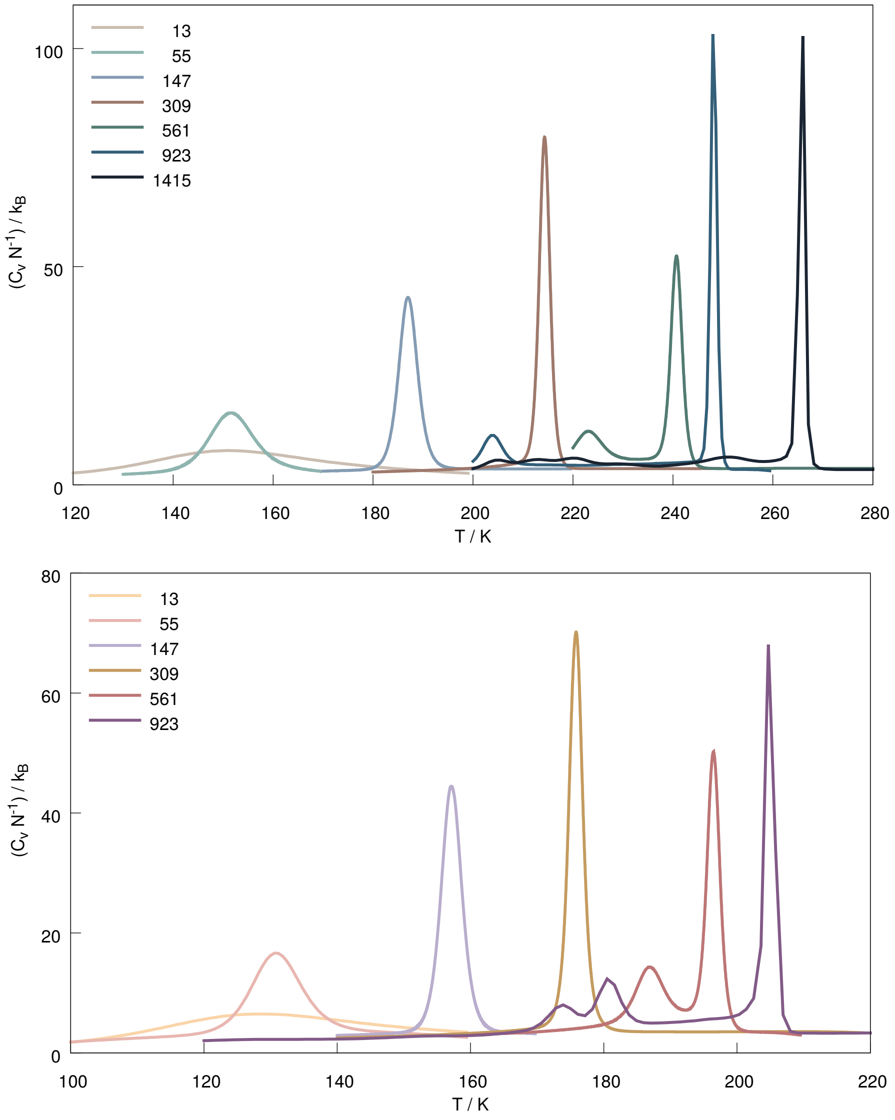

Figure 2. Heat capacities per atom as a function of temperature for simulations at constant volume with the Mackay icosahedral clusters up to 1415 atoms for oganesson at the scalar relativistic (SR) level of theory. Top, heat capacity curves obtained considering 2-body interactions only, using the ELJ potential. Bottom, obtained heat capacities considering 2- and 3-body interactions, computed with the ELJ and EAT potentials respectively.

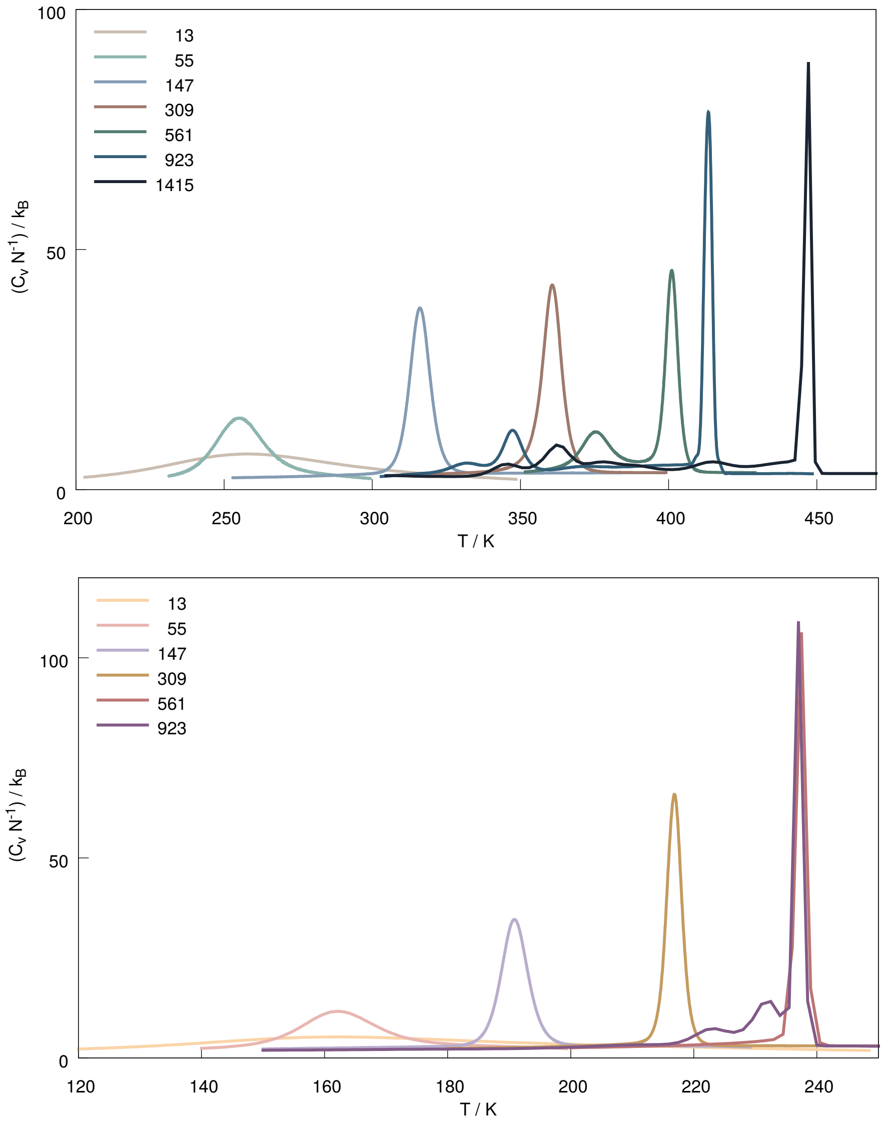

Figure 3. Heat capacities per atom as a function of temperature for simulations at constant volume with the Mackay icosahedral clusters up to 1415 atoms for oganesson at spin-orbit corrected (RX2C) levels of theory. Top, heat capacity curves obtained considering 2-body interactions only, using the ELJ potential. Bottom, obtained heat capacities considering 2- and 3-body interactions, computed with the ELJ and EATM potentials respectively.

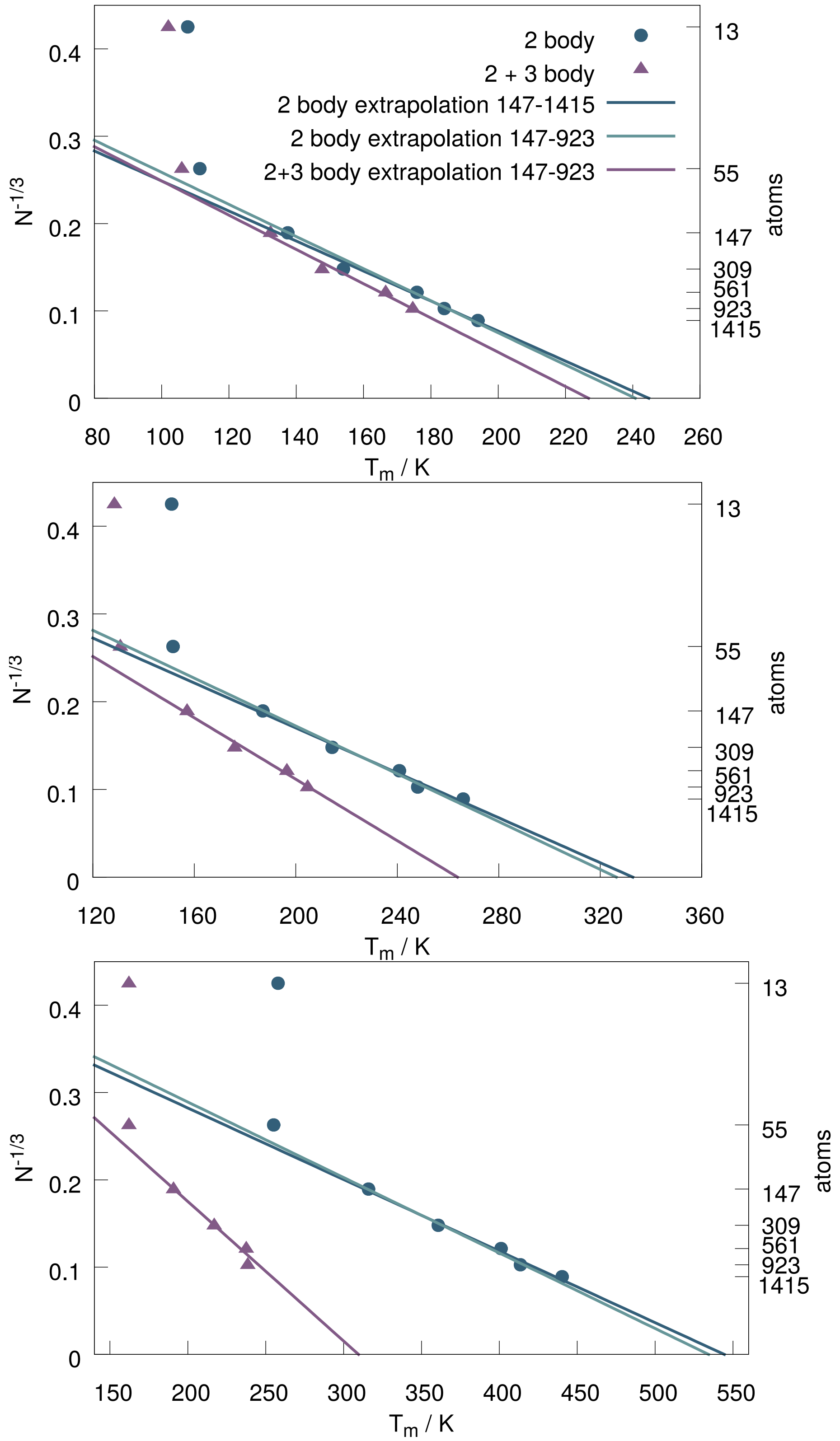

Figure 4. Extrapolated melting temperatures for oganesson at the non-relativistic (NR), scalar relativistic (SR) and spin-orbit (RX2C) levels of theory, obtained using two-body ELJ interactions (circles) and three-body-corrected EAT values (triangles). The blue line corresponds to the linear fit of the melting temperatures of the N= 147-1415 ELJ clusters, light blue to the melting temperatures of the N= 147-923 ELJ clusters and the purple line corresponds to the linear fit through the N= 147-923 clusters that include 3-body EAT corrections.

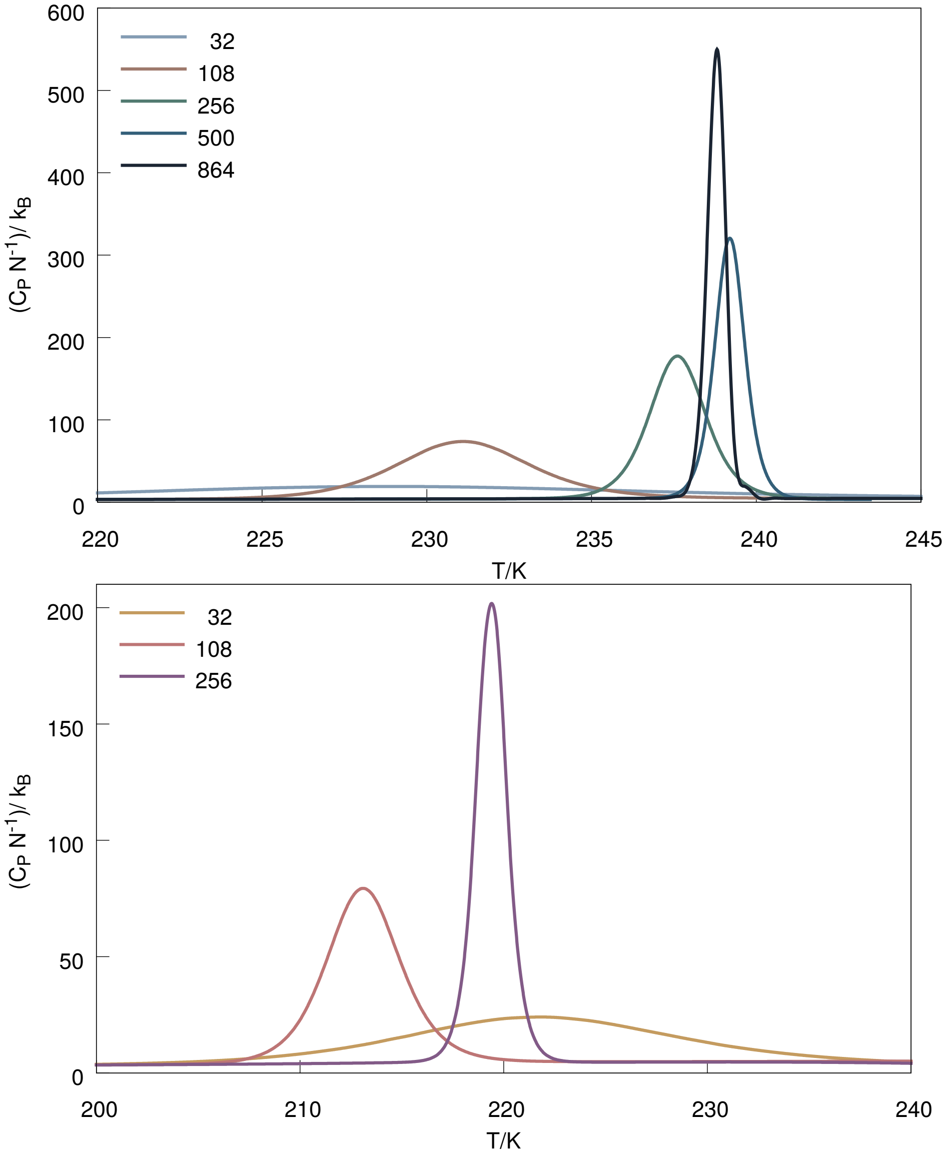

Figure 5. Heat capacities per atom as function of simulation temperature for simulations with periodic boundary conditions and constant pressure for oganesson at non-relativistic (NR) level of theory. Top, heat capacity curves obtained considering two-body ELJ interactions only. Bottom, obtained heat capacities considering two- and three-body interactions, computed with the ELJ and EATM potentials respectively.

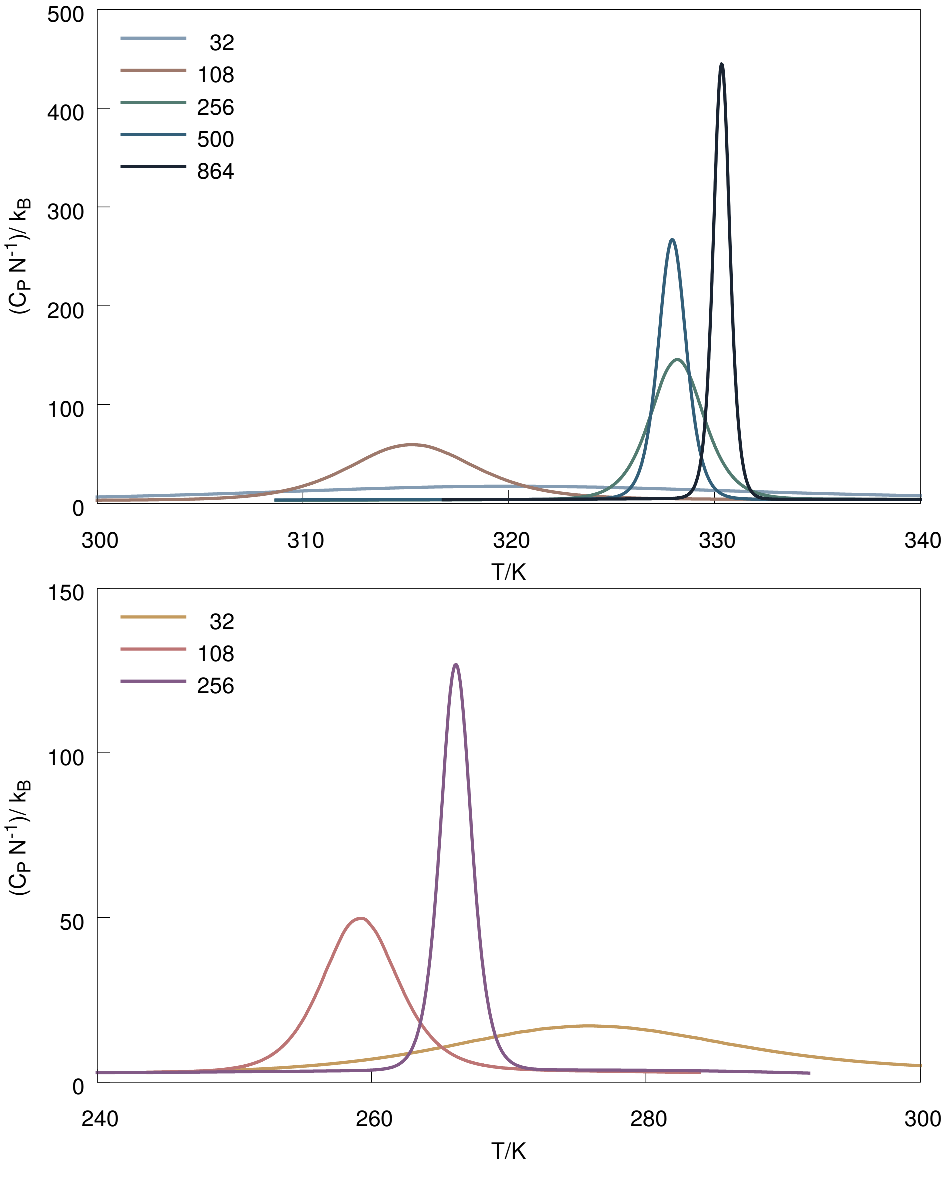

Figure 6. Heat capacities per atom as function of simulation temperature for simulations with periodic boundary conditions and constant pressure for oganesson at scalar relativistic (SR) level of theory. Top, heat capacity curves obtained considering two-body ELJ interactions only. Bottom, obtained heat capacities considering two- and three-body interactions, computed with the ELJ and EATM potentials respectively.

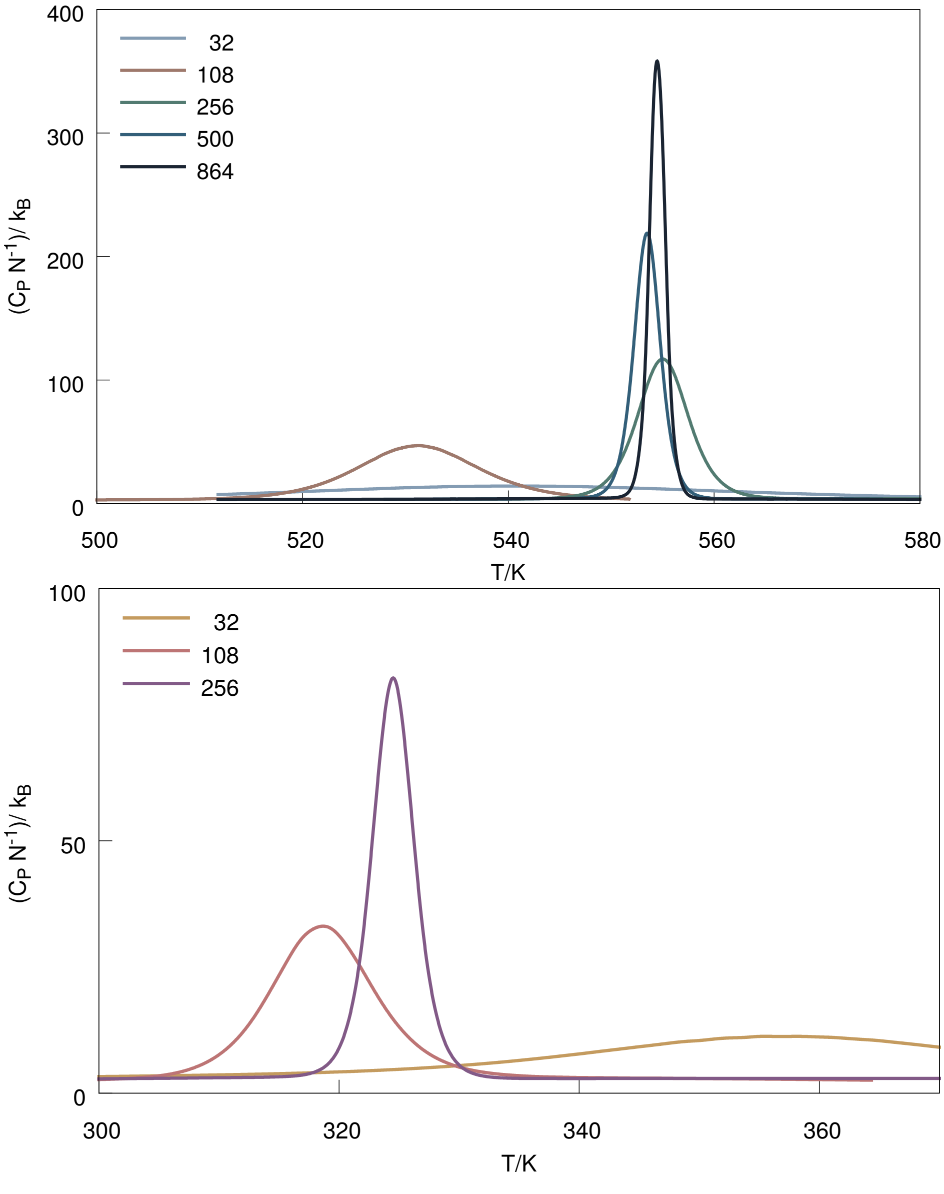

Figure 7. Heat capacities per atom as function of simulation temperature for simulations with periodic boundary conditions and constant pressure for oganesson at spin-orbit corrected (RX2C) level of theory. Top, heat capacity curves obtained considering two-body ELJ interactions only. Bottom, obtained heat capacities considering two- and three-body interactions, computed with the ELJ and EATM potentials respectively.

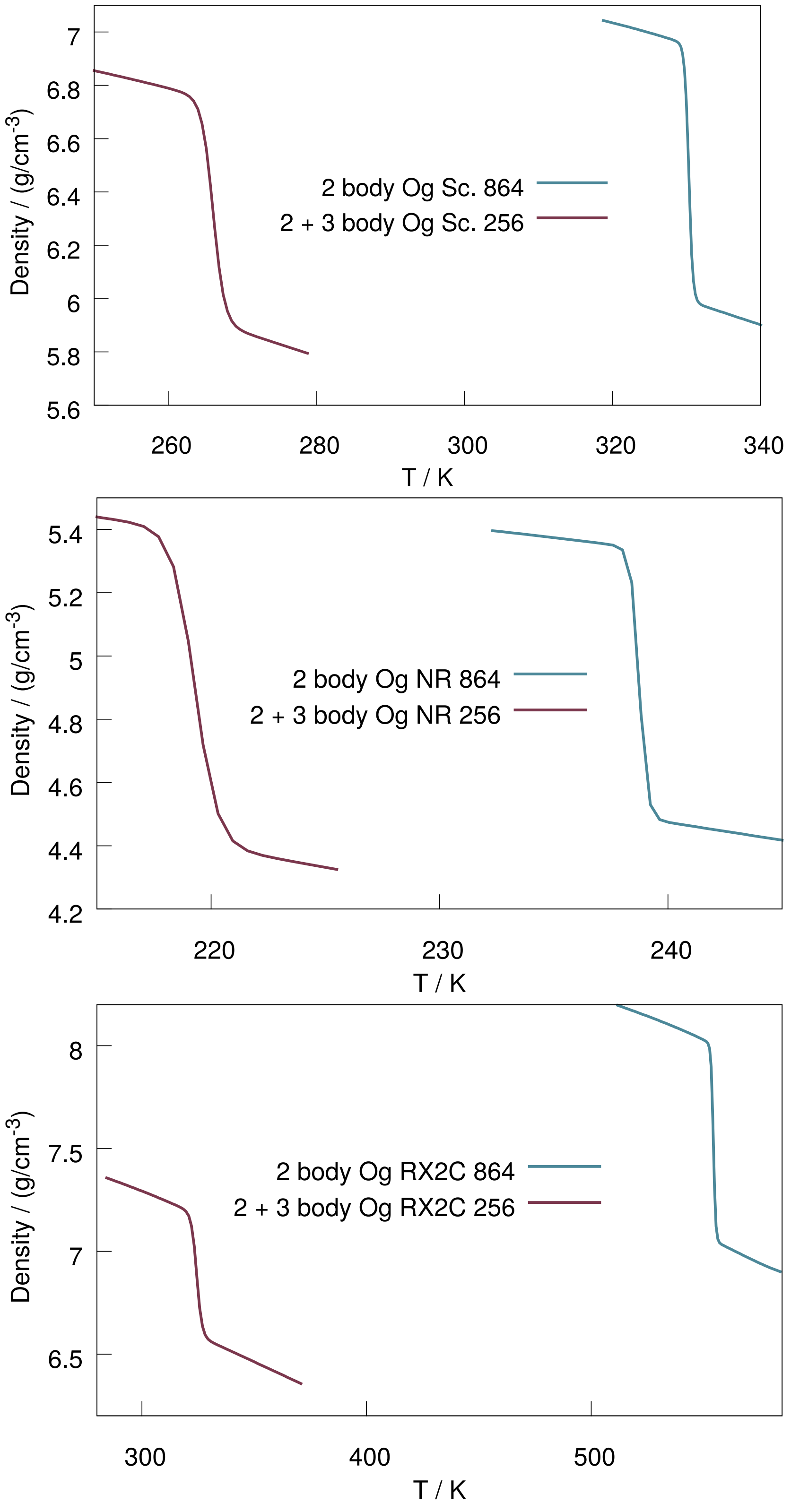

Figure 8. Density of the simulated noble gases. In blue the densities for MC simulation including 2-body ELJ interactions only, in red the densities obtained including 2-body ELJ and 3-body EATM interactions.

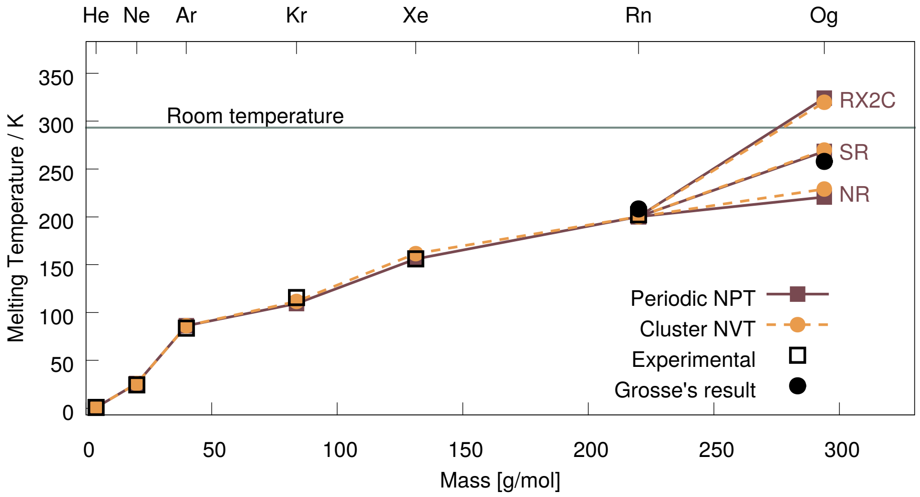

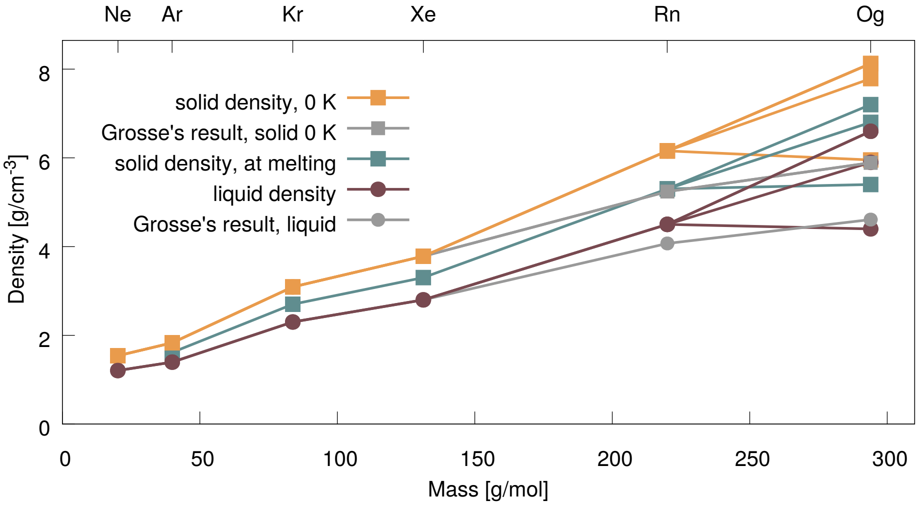

Figure 9. Top, solid densities at 0 K obtained from our solid state calculations and solid and liquid densities at the melting point obtained with the PTMC periodic simulations plotted against their respective atomic mass. Grosse's estimated results are marked with open circles.
Bottom, melting temperatures obtained from the PTMC periodic (squares) and cluster (circles) simulations plotted against the atomic mass. Experimental melting points are marked with open squares, Grosse's predicted values are marked with circles.

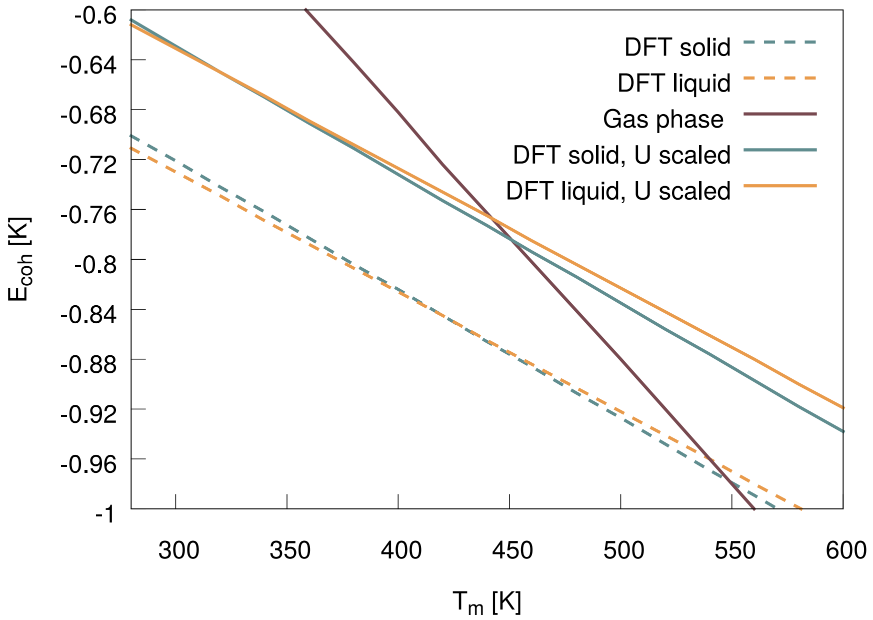

Figure 10. Solid, liquid and gaseous Gibbs free energies plotted against the temperature with the melting and boiling points marked
